# Current and future concepts for the generation and application of genetically engineered CAR-T and TCR-T cells

**DOI:** 10.3389/fimmu.2023.1121030

**Published:** 2023-03-06

**Authors:** Michael Hiltensperger, Angela M. Krackhardt

**Affiliations:** ^1^ German Cancer Consortium (DKTK), partner site Munich and German Cancer Research Center (DKFZ), Heidelberg, Germany; ^2^ IIIrd Medical Department, Klinikum rechts der Isar, School of Medicine, Technical University of Munich, Munich, Germany; ^3^ Center for Translational Cancer Research (TranslaTUM), School of Medicine, Technical University of Munich, Munich, Germany; ^4^ Bavarian Cancer Research Center (BZKF), Erlangen, Germany

**Keywords:** genetically engineered T cells, cancer immunotherapy, adoptive cell therapy (ACT), CAR-T cells, TCR-T cells, CAR (chimeric antigen receptor), TCR (T cell receptor)

## Abstract

Adoptive cell therapy (ACT) has seen a steep rise of new therapeutic approaches in its immune-oncology pipeline over the last years. This is in great part due to the recent approvals of chimeric antigen receptor (CAR)-T cell therapies and their remarkable efficacy in certain soluble tumors. A big focus of ACT lies on T cells and how to genetically modify them to target and kill tumor cells. Genetically modified T cells that are currently utilized are either equipped with an engineered CAR or a T cell receptor (TCR) for this purpose. Both strategies have their advantages and limitations. While CAR-T cell therapies are already used in the clinic, these therapies face challenges when it comes to the treatment of solid tumors. New designs of next-generation CAR-T cells might be able to overcome these hurdles. Moreover, CARs are restricted to surface antigens. Genetically engineered TCR-T cells targeting intracellular antigens might provide necessary qualities for the treatment of solid tumors. In this review, we will summarize the major advancements of the CAR-T and TCR-T cell technology. Moreover, we will cover ongoing clinical trials, discuss current challenges, and provide an assessment of future directions within the field.

## 1 Introduction

Cancer immunotherapies, specifically immune checkpoint inhibition (ICI), have shown high efficacy in the treatment of an increasing number of cancer entities (1). However, a significant portion of patients does not respond to ICI and there is an unmet medical need in these patients for alternative treatment options. One promising new avenue for the treatment of refractory tumors is the field of adoptive T-cell therapy with hundreds of ongoing clinical trials (2). This cell-based personalized therapy either utilizes the patients’ own tumor-infiltrating lymphocytes (TIL) or uses genetically modified T cells with engineered chimeric antigen receptors (CAR) or T cell receptors (TCR) to target and kill tumor cells. Its most prominent form is CAR-T cell therapy, which shows great efficacy in certain hematological cancers and several CAR-T cell therapies have already been approved by the Food and Drug Administration (FDA) for the treatment of blood cancers (2). CAR designs have undergone many iterations in a short amount of time and led to impressive improvements over previous generations of CAR formats. However, their effectiveness in the treatment of solid tumors so far is limited. On the other hand, TCR-T cell therapies have not yet been approved for clinical application but are currently tested in early clinical trials (2). TCR-T cells are not restricted to surface antigens and are more sensitive regarding the level of antigens on the tumor cell compared to CAR-T cells (3). Their dependence on a specific human leukocyte antigen (HLA) composition of the patients, however, restricts this therapy to specific patient populations. Here, we will give an overview of the vast field of CAR-T and TCR-T cell therapy from the manufacturing processes and their impact on the antitumor activity of the T cell product to the feasibility of potential strategies to improve the treatment of refractory tumors.

## 2 Design of engineered CAR and TCR formats

**Figure 1:**
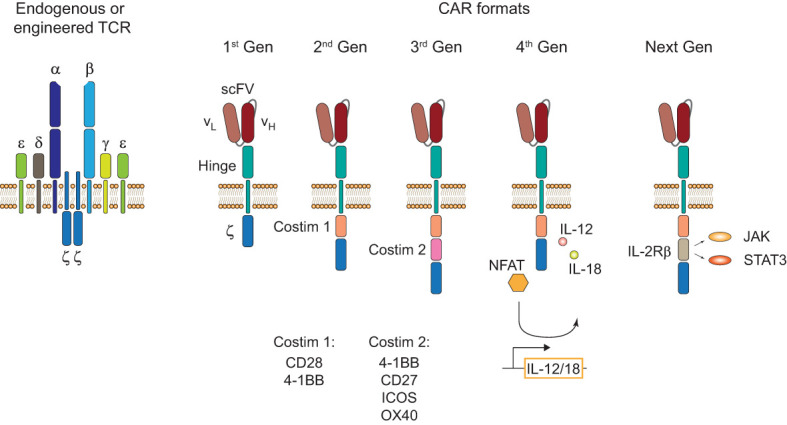
TCR and CAR formats. Structure of an endogenous or genetically engineered T cell receptor (TCR)-CD3 complex (left). Generations of chimeric antigen receptors (CARs) and their structural differences (right). First-generation (1^st^ Gen) CARs only consist of a single-chain variable fragment (scFV), a hinge domain/spacer, and an intracellular CD3ζ signaling domain. Second- (2^nd^ Gen) and third-generation (3^rd^ Gen) CARs include one or two costimulatory domains, respectively. Fourth-generation (4^th^ Gen) CARs or T cells redirected for antigen-unrestricted cytokine-initiated killing (TRUCKs) include a transgenic expression cassette for nuclear factor of activated T-cells (NFAT)-mediated transgene expression. Next-generation (Next Gen) CARs include a truncated intracellular domain of cytokine receptors with a STAT-binding motif for JAK/STAT signaling.

Endogenous and engineered TCRs recognize peptide-HLA complexes on target cells representing the antigen of interest. Engineered TCRs in general do not deviate from the classical TCR structure of an α-/β-chain heterodimer and are able to form functional TCR-CD3 complexes (**Figure 1**, left). Upon antigen recognition the two intracellular CD3ζ domains induce downstream TCR signaling. In contrast, CARs are designed as single molecules that consists of a single-chain variable fragment (scFV), a hinge domain, a transmembrane domain, and intracellular costimulatory signaling domains (**Figure 1**, right). Antigen recognition is facilitated by the scFV, a fusion protein of the light and heavy chain variable regions of an antibody that are connected by a peptide linker (4). Contrary to engineered or endogenous TCRs, CARs cannot assemble CD3 complexes and antigen recognition of surface antigens by the scFV is HLA-independent. First-generation CARs proved the feasibility of the concept by showing that coupling to an intracellular CD3ζ domain is sufficient for downstream signaling upon antigen recognition (5). The next iteration to the format included a costimulatory signaling domain, CD28 or 4-1BB, proximal to the membrane to incorporate both primary and costimulatory signaling with increased IL-2 production (6). To enhance antitumor activity and potentially increase persistence of CAR-T cells, a second costimulatory domain was added in third-generation CARs (7, 8). There is a number of third-generation CAR-T cells currently tested in clinical studies (NCT03676504 (9); NCT04049513 (10)) that showed good safety profiles and will evaluate their persistence in patients with CD19^+^ malignancies. One interesting finding in support of this comes from a phase I clinical trial (NCT01853631) that observed greater expansion and longer persistence of CD19 third-generation (CD28 and 4-1BB) CAR-T cells compared to second-generation (CD28) cells when infused simultaneously in patients with r/r NHL (11). A new concept was applied in fourth-generation CARs or T cells redirected for antigen-unrestricted cytokine-initiated killing (TRUCKs). TRUCKs combine the introduction of a CAR with a transgenic expression cassette consisting of synthetic nuclear factor of activated T-cells (NFAT) response elements with an IL-2 minimal promoter and transgenes. CD3ζ-mediated signaling ultimately leads to the phosphorylation of NFAT, its translocation into the nucleus, and the expression of the transgenes (12). Because the TRUCK concept is dependent on CD3ζ-mediated NFAT translocation, it is applicable not only for CAR-T but also for TCR-T cells (13). The most common transgenic proteins for this approach are IL-12 and IL-18 (14–17) but many other cytokines and enzymes are currently explored (12). The TRUCK concept is of particular interest for the treatment of solid tumors by combining T cell-mediated killing with immune modulation of the tumor microenvironment (TME) through the secretion of cytokines. IL-12 and IL-18 secretion in the TME might augment the antitumor cascade by attracting and activating macrophages and NK cells (13). Since CAR constructs do not have a specific domain for cytokine-mediated signaling (also known as signal 3), novel developments include CARs with a truncated intracellular domain of cytokine receptors (e.g. IL-2 receptor β (IL-2Rβ) domain) and a STAT3-binding motif to induce JAK/STAT signaling (18). This approach prevented terminal differentiation *in vitro* and showed increased persistence and antitumor activity in preclinical tumor models compared to second-generation CAR-T cells (18) but this format likely needs further evaluation to prove its translational potential.

CAR-T cells have inherently lower antigen sensitivity compared to canonical T cells and tonic CAR signaling has been associated with CAR-T cell exhaustion (3, 19). This is likely at least in part due to the differences in signaling modalities of a TCR-CD3 complex that contains 10 immunoreceptor tyrosine-rich activation motifs (ITAMs) compared to conventional CARs that only contain three ITAMs (20). To engage the endogenous CD3 signaling complex with antibody-mediated antigen recognition, a double-chain chimeric receptor, termed synthetic TCR and antigen receptor (STAR), fused the constant α and β domain to the light and heavy chain variable regions of an antibody has been developed (21). Upon antigen recognition, STAR has been demonstrated to provide TCR-like signaling with superior antigen sensitivity and antitumor activity compared to a second-generation CAR-T cell in solid tumor mouse models. This might be an interesting new design, however, a potential risk for increased on-target off-tumor toxicity due to its enhanced antigen sensitivity could limit its clinical application and needs to be investigated.

## 3 Manufacturing of genetically engineered T cells

**Figure 2:**
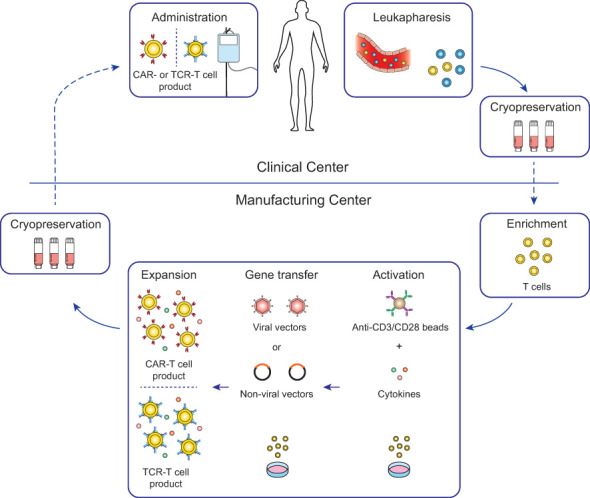
Workflow for conventional CAR-T and TCR-T cell therapy. For most conventional CAR-T and TCR-T cell therapies, autologous patient-derived leukocytes are collected by leukapheresis at clinical centers (top) and after cryopreservation shipped to manufacturing centers (bottom). Enriched T cells are genetically engineered with either CARs or TCRs and after expansion cryopreserved and shipped to the clinical centers for infusion into the patients. Lymphodepleting chemotherapy is generally recommended in the week before infusion to increase engraftment of the genetically engineered T cells (26).

Genetically engineered T cell products were initially developed in open self-operated bioreactors that are common in academic institutions. However, these systems require well-trained staff and rigorous hygiene monitoring to avoid contaminations. Therefore, with the clinical successes of CAR-T cell therapies, the production has shifted not only in industrial facilities but also in academic institutions more and more to closed and semi- or fully-automated platforms (22–25). Most advances to the manufacturing platforms for CAR-T cells are likely transferable for the production of TCR-T cells with some modifications to the protocols. Therefore, TCR-T cell therapies greatly benefit from the innovations in the ACT field which were set in motion by the clinical approval of CAR-T cell therapies. The general workflow of CAR-T and TCR-T cell therapy is depicted in **Figure 2** and will be summarized in this chapter in detail.

### 3.1 Cell collection and handling

For the generation of genetically engineered T cells, leukocytes are collected either from patients (autologous) or healthy donors (allogeneic). Notably, all FDA-approved CAR-T cell therapies to date are using patient-derived autologous cells but there is a number of clinical trials investigating the use of donor-derived allogeneic cells (2). Leukapheresis is the method of choice for the collection of leukocytes due to its availability at health care centers, patient tolerability, and its high yield of T cells for manufacturing (27, 28). Collected cells are either used fresh for direct manufacturing or more commonly cryopreserved for later handling. Cryopreservation takes place at the clinical center or in some cases at the manufacturing center. Although, cryopreservation has an impact on cell viability, on-site manufacturing of genetically engineered T cells is often not feasible – with the exception of a few academical clinical studies (NCT03676504 (9)) – and CAR-T cell generation can be achieved with frozen cells (29). There are differences of the cryopreservation procedure across clinical and manufacturing centers for different T cell products, regarding freezing media composition and durations, but their impact on the final product quality has not yet been comparably assessed.

### 3.2 Impact of starting cell composition on antitumor immunity

The cellular composition of the starting material is paramount for the success to engineer a functional CAR-T cell product with long-lived antitumor properties. Enrichment of T cells from the leukapheresis product can be achieved with magnetic separation beads and all approved CAR-T cell therapies to date either use CD4^+^ and CD8^+^ T cells in a combined or a separate culture setting. Thus, CAR-T cell therapies are not limited to only generating CD8^+^ CAR-T cell responses but also utilize CD4^+^ CAR-T cells to improve their antitumor response in a synergistic fashion (30, 31). CD4^+^ T cells show more plasticity compared to CD8^+^ T cells and are comprised of T helper (Th) subsets and regulatory T (Treg) cells (32). Perturbation of CD4^+^ subsets and generation of Treg cells in particular might have an impact on the clinical response. In a recent study, the expansion of CAR-Treg cells has been associated with resistance to CD19 CAR-T cell therapy (33). Therefore, differentiation of CD4^+^ T cells to an “optimal” antitumor phenotype and limited generation of CAR-Treg cells during manufacturing might be essential to improve ACT. Separate manufacturing and administration of CD4^+^ and CD8^+^ T cells is already applied for the CD19 CAR-T cell therapy Breyanzi to reduce variability between the CD4^+^ and CD8^+^ CAR-T cell composition and to administer it in a dose-defined manner (34). In a clinical trial of a CD22 CAR-T cell therapy in children and young adults with CD22^+^ B-cell neoplasms, the change of the manufacturing protocol to CD4^+^ and CD8^+^ T cell selection improved manufacturing feasibility and reduced variability, however, this led to increased inflammatory toxicities and warranted dose de-escalation (35). The overall complete remission rate was still very high with 70% but may indicate CAR-T cell-induced toxicities based on the ratio of CD4^+^/CD8^+^ cells in the CAR-T cell product. In some cases separate manufacturing of CD4^+^ and CD8^+^ CAR-T cells could compromise CAR-T cell expansion. This has been the case in a third-generation CD20 CAR-T cell therapy where changing from CD4^+^/CD8^+^ selection to a combined culture setting improved manufacturing feasibility and clinical response rates (36). Moreover, enrichment and differentiation of specific memory T cell subsets, like multipotent T memory stem (TSCM) cells, may improve the antitumor responses of CAR-T cells (37, 38). This might be due to a certain level of stemness of the T cells that comes with higher T cell persistence and less susceptibility to exhaustion (39–41). Overall, the impact of the cellular composition during manufacturing on the antitumor efficacy of T cell therapies still needs to be compared in future clinical studies.

### 3.3 Activation conditions during manufacturing

T cells are activated for efficient gene transfer and expansion which is commonly achieved by using anti-CD3/CD28 paramagnetic beads for viral transduction (42). However, this approach was reported to favor the expansion of CD4^+^ T cells over CD8^+^ T cells in non-enriched products (43) and could lead to even inefficient expansion of CD8^+^ T cells in some cases (44). This is likely due to the importance of CD28-mediated signaling in CD4^+^ T cells while 4-1BB costimulation is superior for the expansion of CD8^+^ memory T cells (44). To overcome this, current protocols have introduced cytokine cocktails in addition to anti-CD3/CD28 beads to support the expansion and to skew the differentiation into a phenotype with inherent good antitumor characteristics (45–49). For example, the cytokine IL-2 is used in standard protocols for its mitogenic effects on T cells and its potential benefits on T cell effectiveness in the context of tumor immunity (50).

In addition, the activation conditions and strength of the stimuli during manufacturing could also determine if the genetically engineered T cells are prone to exhaustion upon encountering their cognate antigen on the tumor cells. Soluble anti-CD3 antibodies together with mononuclear cells have been shown to result in a similar expansion efficacy of CD8^+^ T cells compared to anti-CD3/CD28 beads but induced a less terminally differentiated phenotype as well as less antigen-induced cell death and more expansion in previously activated CD8^+^ T cells (51). Acquisition of terminal differentiated effector functions during manufacturing actually may lead to impaired antitumor immunity *in vivo* (52). Due to the sensitive nature of T cell activation and differentiation and their impact on antitumor immunity and longevity of the genetically engineered T cells, new methods are constantly investigated. The Expamer technology is an interesting new approach for time-controlled initiation and termination by using soluble Strep-Tactin multimers that can be assembled with Twin-Strep-tag conjugated anti-CD3 and anti-CD28 Fab fragments and dissociated by adding non-toxic D-biotin (53). Soluble addition and inactivation of Expamer components for T cell activation is particularly attractive for large-scale production and might be useful to avoid overstimulation and subsequent apoptosis. On the other hand, less rigid surfaces for immobilization of anti-CD3/CD28 binders might be a better alternative since they have been shown to induce higher IL-2 production and expansion of CD4^+^ and CD8^+^ T cells ex vivo (54). Consistent with this, an antigen-presenting cell (APC)-mimetic scaffold that consists of a fluid lipid bilayer supported by mesoporous silica micro-rods and attached with anti-CD3/CD28 antibodies promoted two- to tenfold greater expansion compared to anti-CD3/CD28 paramagnetic beads (55). However, lipid bilayer systems might be less appealing compared to bead-based approaches for activation when it comes to large-scale manufacturing due to their increased technical complexity regarding handling and ease of removal. More studies that compare the effects of different activation conditions on the final T cell product for ACT will be necessary in the future in order to understand their impact on the clinical efficacy.

### 3.4 Gene transfer methods

Viral vectors are used for all approved CAR-T cell therapies and most clinical trials for CAR- and TCR-T cell therapies due to their efficiency for stable gene transfer (56). Lentiviral (LV) and γ-retroviral vectors are the vectors of choice for cell engineering since they can carry larger genetic constructs and integrate the target gene into the genome of the engineered T cells compared to other viral vectors (57, 58). However, streamlining the generation of large quantities of viral vectors for manufacturing is a challenge and ensuring that no residual viral vectors or accidently transduced malignant cells are given to the patients comes with extensive and costly safety testing (59, 60). This presents difficulties for scaling out manufacturing and for making these therapies more affordable to meet the increasing demand for ACT. Therefore, non-viral approaches are currently investigated in early trials. Transposon-based gene delivery approaches, such as Sleeping Beauty (SB) or PiggyBac (PB) transposons, are cheaper and can carry larger genetic constructs compared to viral vectors while still integrating their target gene (61). SB transposition has already been used successfully for the manufacturing of CD19 or SLAMF7 CAR-T cells in early clinical trials without severe toxicity (62–65). In addition, automation of SB transposition was feasible and could be very attractive for large-scale manufacturing (25). Although PB transposition was also successful for the manufacturing of CAR-T cells and even showed an inclination to promote the generation of desired TSCM CAR-T cells (66, 67), a recent clinical trial observed the formation of CAR-T cell lymphoma in two out of ten patients (68, 69). This presented the first cases of malignant lymphoma derived from CAR-T cells and the investigators of the study caution for regular follow-ups of the patients receiving CAR-T cell therapies, especially when new methods for gene transfer are applied (69). The underlying causes for the observed malignant transformation in this study using the PB system is not fully understood yet. Insertional mutagenesis did not seem to be the cause since the pattern of integration of the CAR transgene was comparable to other studies using PB and in line with studies using viral systems (68). However, transcriptional upregulation of surrounding regions by the transgene promotor was observed but how these alterations may be involved in malignant transformation needs to be further addressed. The authors of the study do not think that this finding is an inherent problem with the PB system but rather it might be based on their manufacturing methodology with high-voltage electroporation and high concentration of transposon and transposase (69). Understanding the underlying mechanisms will help to develop safer manufacturing protocols and better safety readouts in the future.

Genome editing is a promising novel approach not only for the generation of genetically engineered autologous but also for “off-the-shelf” allogeneic T cells that might solve manufacturing challenges and excessive cost that are inherent to autologous T-cell therapies. In particular, clustered regularly interspaced short palindromic repeat (CRISPR)-Cas9 has already been used for the generation and clinical application of CAR-T cells with tolerable adverse events in cancer patients (70, 71). Multifactorial genome editing holds great potential for ACT with genetically engineered T cell in the future but will need further optimization and extensive safety monitoring to assess the risks for harmful off-target events. More information about CRISPR-Cas9 genome editing for the generation of engineered T cells is reviewed in (72, 73).

### 3.5 Shortening manufacturing time of genetically engineered T cells

An important avenue for optimizing the manufacturing process of genetically engineered T cells is reducing the manufacturing time. This will reduce the cost and will scale-up manufacturing due to a faster turn-around of the engineered T cells. Most importantly, it might reduce mortality of patients with rapidly progressing cancers by reducing the vein-to-vein time. Standard protocols for CAR-T cell therapy culture CAR-T cells for 11 to 24 days which leads to a high number of harvested CAR-T cells (45–48). Interestingly, reducing the culture time of CD19 CAR-T cells to only 3 days increased their antitumor activity even at a 6-fold lower dose in a human xenograft model of acute lymphoblastic leukemia (ALL) (74). This could be due to an enriched proportion of stem-like T cells in the CAR-T product at reduced culture times. Remarkable manufacturing times haven been achieved with the FastT CAR-T next-day manufacturing platform, that was recently evaluated in a clinical trial for B-cell ALL (NCT03825718 (75)). Next day manufacturing with activation, LV transduction, and without expansion was feasible for all 25 patients with a tolerable safety profile and promising efficacy. Moreover, CD19 FasT CAR-T cells showed less exhaustion and a younger cellular phenotype compared to conventionally manufactured CAR-T cells *in vitro* but evaluation in larger clinical studies is needed. In addition, the T-Charge platform was used for manufacturing of CD19 CAR-T-cells in a phase I study with promising efficacy and safety profile (NCT03960840 (76)). The manufacturing time was less than 2 days and culturing time only took 24 hours. This approach also preserved naïve T and TSCM cells in the final CAR-T cell product which might increase the persistence of the genetically engineered T cells in patients. Rapid manufacturing of CAR-T cells was even demonstrated without activation and expansion within 24 hours and showed improved anti-leukemic activity in mouse xenograft models when compared to their conventionally manufactured counterparts (77). These new approaches seem to have great potential to reduce the vein-to-vein time which would greatly benefit the patients. However, since most of the T cell expansion takes place in the patients, this could mean that adverse events might be more difficult to predict in a temporal fashion upon therapy administration. Therefore, these new methods will have to be thoroughly tested in clinical trials with rigorous monitoring to ensure non-inferiority in efficacy and safety in comparison to standard long-time manufacturing procedures. Improving and standardizing manufacturing protocols for genetically engineered T cells, with every step potentially having an extensive impact on the antitumor activity and safety, is especially difficult since in-depth manufacturing protocols of approved CAR-T therapies are not publicly available. This will present a challenge for the future but overcoming this roadblock by greater exchange could fuel innovation and accessibility of these groundbreaking new therapies.

## 4 Targeted gene delivery *in vivo*

Due to the high cost of ex vivo T cell manufacturing, long vein-to-vein times which are problematic for highly progressive cancers, and the risk of manufacturing failure, targeted *in vivo* programming of T cells could be a viable alternative. DNA-carrying nanoparticles have been demonstrated to translocate into the nucleus of T cells followed by CAR expression in the targeted T cells (78). In addition, *in vivo* administration of mRNA nanocarriers for the delivery of antitumor CARs or antiviral TCRs showed transient expression in T cells and comparable disease regression in mice compared to their ex vivo manufactured counter parts (79). These carrier systems are inexpensive and can be manufactured in large scale for broader distribution but their safety profile and efficacy regarding long-term disease remission is still largely unclear. The transient expression will likely reduce certain safety concerns, such as programing of malignant cells by accident and rendering them resistant to therapy (59) or the formation of CAR-T lymphoma (69). However, the lack of long-term T memory formation could hinder the efficacy of the treatment and might lead to earlier relapses compared to ex vivo manufactured T cells. Thus, their efficacy compared to more established approaches, such as bispecific T cell engagers (BiTEs), might not be superior.

Viral approaches have also been tested for long-term programing of T cells *in vivo*. Adeno-associated virus (AAV) vector achieved introduction of CARs in humanized mice and resulted in tumor regression (80). The AAV-based gene therapy LUXTURNA for the treatment of patients with *RPE65*-mediated inherited retinal dystrophy (81) is the first of its kind that received FDA approval, which could increase the interest for AVV vectors also for the clinical application of T cell programming *in vivo* for the treatment of cancer. A strategy for targeted *in vivo* engineering of a CD19 CAR was shown in a study using CD8α-chain targeted LVs (82). Although the specificity for CD8 T cells was good with this approach, NK and NKT cells also showed transduction for the CD19 CAR since they are also expressing the targeted CD8α-chain. LV targeting to CD3^+^ T cells was also feasible by using bispecific antibody tandem fragments that bind the mutant E2 glycoprotein on Sindbis pseudotyped lentiviral vector (SINV-LV) and CD3 on T cells, achieving specific *in vivo* introduction of a CD19 CAR into T cells with good antitumor efficacy in a human B cell tumor xenograft model (83). Despite the early preclinical and clinical successes of viral *in vivo* transduction for cell engineering and gene therapy, this strategy comes with much higher safety risks compared to ex vivo manufacturing of genetically engineered T cells and it remains to been seen if it can meet the high safety requirements.

## 5 Specific requirements of gene transfer techniques for TCR-T cells

**Figure 3:**
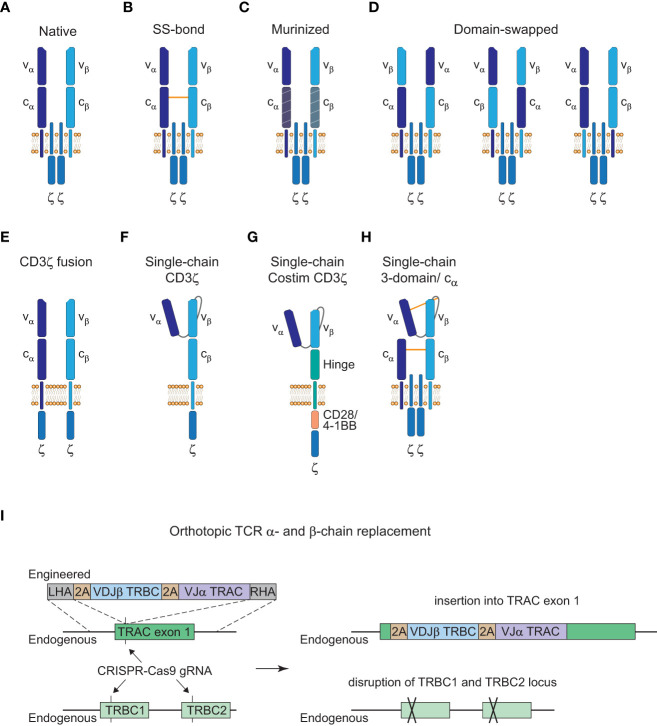
Strategies to avoid TCR mispairing. **(A)**, Native structure of a T cell receptor (TCR) α:β heterodimer (CD3ε, CD3δ, and CD3γ subunits are not shown here). **(B)**, Engineered TCR α:β heterodimer with an extra disulfide (SS) bond between the constant α and β domain. **(C)**, Engineered TCR α:β heterodimer with murine constant α- and β-domain. **(D)**, Engineered TCR α:β heterodimer with α- and β-domain swapping of the variable (left), constant (middle) or transmembrane (right) domains. **(E)**, Engineered TCR α:β heterodimer with a fused intracellular CD3ζ domain. **(F)**, Engineered single-chain TCR with a fused intracellular CD3ζ domain. **(G)**, Engineered single-chain TCR with a hinge domain instead of a constant and transmembrane β domain and inclusion of a costimulatory (Costim) and a CD3ζ domain. **(H)**, Engineered 3-domain single-chain TCR with an extra SS bond between the constant α and β domain and the variable α domain and a linker residue in close proximity to the variable β domain. A constant α domain is co-expressed with the 3-domain single-chain TCR. **(I)**, Orthotopic TCR α- and β-chain replacement with CRISPR-Cas9 gene editing. Engineered TCR construct is introduced into exon 1 of TRAC with a left homology arm (LHA) and a right homology arm (RHA). The endogenous TRAC locus is disrupted by the insertion of the engineered TCR construct and the endogenous TRBC1/TRBC2 gene locus is disrupted with another guide RNA (gRNA).

Many advances of the manufacturing processes for CAR-T cell therapies are likely transferable to TCR-T cell therapies. However, one specific problem for the generation of TCR-T cells is that introduction of an engineered TCR into a T cell can cause mispairing of the engineered α- or β-chains with the endogenous chains. This presents an unpredictable risk since mispaired TCRs have unknown reactivity that never went through thymic selection and could result in the formation of TCRs against self-peptides and thereby autoimmunity or graft-versus-host disease (GvHD) (84, 85). Although earlier clinical trials with TCR-T cells that retained their endogenous TCR did not observe GvHD (86), ways to prevent the safety risk of mispairing would be beneficial. Therefore, a number of strategies have been developed to avoid mispairing events with varying success as illustrated in **Figure 3**.

Insertion of an extra disulfide bond into the constant domains, murinization of the constant domains or domain swapping all led to the reduction of mispairing events but could not prevent it completely (**Figures 3B–D** (85, 87, 88)). Only CD3ζ fusion chains or single-chain TCR (scTCR) constructs abolished mispairing but do not form a TCR complex with the endogenous CD3γ, δ, and ε subunits (**Figures 3E, F** (89, 90)). Further elimination of the constant β domain and the addition of an intracellular costimulatory CD28 or 4-1BB domain has been reported which resembles the CAR structure and signaling modalities (**Figure 3G** (91, 92)). However, this altered structure has reduced sensitivity compared to the native TCR-CD3 complex as is the case for conventional CARs (3). Therefore, a more recent scTCR scaffold tried to incorporate the assembly of the native CD3 complex to harness the benefits of classical TCR signaling (**Figure 3H** (93)). This 3-domain scTCR consists of a vα-linker-vβ fragment fused to the cβ-domain and utilizes co-expression of the cα-domain with very little mispairing occurring. In addition, insertion of a disulfide bond between the vα-domain and a linker residue in close proximity to the vβ-domain was sufficient to prevent residual mispairing (93). Although this might provide a safe alternative for the introduction of engineered TCRs without the danger of mispairing events, the design of stable scTCRs could present a technical challenge for a number of TCRs. Stability engineering might be a potential solution for this limitation, since distinct regions in the vα- and vβ-domains have already been shown to be critical for surface expression and stability of scTCRs (94, 95).

Alterations from the native TCR structure could lead to a higher immunogenicity and reduced persistence of genetically engineered T cells if the constructs deviate greatly from the native format. Thus, another approach to avoid mispairing without altering the TCR structure is to knock-out the endogenous α- and β-chain. Orthotopic TCR α- and β-chain replacement (OTR) was done by CRISPR-Cas9 genome editing and was recently used in a phase I clinical trial to achieve endogenous TCR replacement with neoantigen-specific TCRs (neoTCRs) in 16 patients with refractory solid cancers (**Figure 3I** (70, 96, 97)). Insertion of the engineered TCR construct into exon 1 of the TRAC locus disrupts the endogenous α-chain and CRISPR-mediated knockout of the TRBC locus causes disruption of the endogenous β-chain. Another advantage of OTR is that there is no competition for the CD3 subunits with the endogenous TCR to form core TCR-CD3 complexes (98). However, cell-surface expression of the introduced TCRs can be inefficient in some cases with this editing approach. In the recent trial mentioned above, out of the 37 neoTCRs generated for 16 patients, cell-surface expression of neoTCR positive cells ranged from 1.9 to 46.8% of the live cell product (97). This might potentially be a matter of protocol optimization since changes to the medium formulation and the electroporation device increased the knock-in efficiency from 13.4% to 23% in the same study. Moreover, chromosomal aberrations at the chromosome 7 and 14 target sites were observed and are an indication for potential *TRAC : TRBC* translocations. Off-target editing and on-target mutagenesis present a major concern for CRISPR-mediated approaches since they could lead to functional alterations or even malignant transformation of the edited cells (99, 100). Close monitoring of patients will be crucial for these newer approaches and further efforts will be necessary to understand and to reduce unwanted DNA aberrations for clinical application (101).

## 6 Genetically engineered allogeneic T cells

To date, all approved CAR-T cell therapies are utilizing patient-derived autologous T cells. However, since only heavily pretreated patients are applicable for immunotherapy with CAR-T cells, their T cell compartment is often compromised (27). This can result in reduced fitness or even manufacturing failure of the autologous CAR-T cell product (102, 103). Moreover, personalized production for each patient is challenging and very costly (as described above) and does not allow for mass production to meet the increasing demand for ACT. “Off-the-shelf” allogeneic T cells could be an approach to overcome these limitations. Because unaltered allogeneic T cells could lead to GvHD but also elimination of the genetically engineered T cells in the patient, a number of strategies have been developed to reduce these risks.

For the treatment of relapsed or refractory (r/r) B-cell ALL an allogeneic CD19 CAR- (NCT02808442 and NCT02746952) and a CD19/CD22 dual-targeting CAR-T cell therapy (NCT04154709) have shown manageable safety profiles and anti-leukemic activity (104, 105). CRISPR-Cas9 genome editing was used to disrupt the *TRAC* and *CD52* gene locus allowing severe lymphodepletion with alemtuzumab prior to adoptive T cell transfer to reduce the risk for elimination of allogeneic engineered T cells by the host. In another first-in-human phase I clinical trial (NCT04637763), early positive results of an allogeneic CD19 CAR-T cell therapy for the treatment of r/r B cell non-Hodgkin lymphoma (B-NHL) were reported (106). Cas9 and CRISPR hybrid RNA-DNA (chRDNA) guides were used for reduced off-target editing (107) to introduce the CD19 CAR into the *TRAC* gene locus and disrupt it in the process (108). In addition, PD-1 was knocked out with the aim to improve persistence and antitumor activity of the genetically engineered T cells. No GvHD was observed and the therapy was generally well tolerated with promising clinical response rates. Another targeted approach is the use of a TRAC-specific ARCUS nuclease for site-specific introduction of the construct and disruption of the endogenous TCR to avoid GvHD (109). An allogeneic CD19 CAR-T cell therapy using this editing approach showed promising results in a Phase I/IIa clinical trial (NCT03666000) for the treatment of r/r B-NHL and B-cell ALL and could potentially be used for the treatment of relapsed patients with lymphomas that previously received autologous CAR-T cells. All 11 patients showed an objective response rate (ORR) after six months but no reported GvHD.

Genome editing also allows for the generation of fratricide-resistant CAR-T cells. This has been shown in a phase I clinical trial (NCT04538599) for a CD7 CAR T cell therapy for the treatment of T-cell lymphoma and CD7-expressing acute myeloid leukemia (AML) (110). Because CD7 is also expressed on normal T cells, CD7 was knocked-out to avoid fratricide and a number of additional edits (knock-out of TCR and HLA-II, knock-in of an NK cell inhibitor) were performed to avoid GvHD of the allogeneic CAR-T cell product.

Allogeneic TCR-T cells were also successfully tested in an investigator-initiated Phase I/II clinical trial (NCT01640301) for patients with AML receiving allogeneic hematopoietic cell transplantation (HCT) with high risk of relapse (111). Epstein-Bar virus (EBV)-specific CD8^+^ T cells from the HCT donor were transduced with a TCR that recognizes the AML-associated intracellular antigen Wilms’ Tumor Antigen 1 (WT1). Patients with HLA-A*0201 expression that received an allogeneic HCT and had no detectable disease at day 28 post-HCT were given engineered WT1-specific T cells prophylactically. All 12 patients showed relapse-free survival at a median of 44 months and compared very favorable to a similar risk group of 88 patients with 54% relapse-free survival. These results encourage the use of allogeneic consolidating ACT as a strategy for the prevention of AML relapses after HCT. In addition, allogenic HA-1-specific TCR-T cell therapy for the treatment of HLA-A*0201 positive patients with r/r ALL after allogeneic HCT is currently explored in a dose-escalation study (NCT03326921).

Preliminary results from early clinical studies with allogeneic engineered T cells that we described here are encouraging regarding their efficacy and risk of GvHD, however, this approach is still in its early stages and we will need to wait out larger clinical studies to assess its clinical value in the future.

## 7 Current challenges and potential strategies to improve CAR-T and TCR-T cell therapies

**Figure 4:**
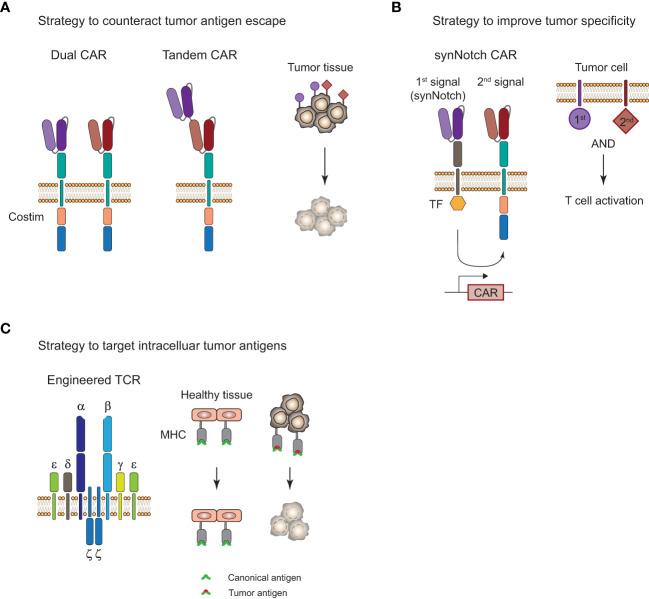
Strategies to improve adoptive T-cell therapy for the treatment of tumors. **(A)**, Strategy to overcome tumor antigen escape by using bi-specific dual or tandem CAR-T cells. **(B)**, Strategy for increased tumor specificity and reduced off-tumor toxicity with a synthetic Notch (synNotch) receptor. Encounter of a primary antigen (purple) leads to translocation of a transcription factor (TF) into the nucleus and expression of a CAR that recognizes a secondary antigen (red) on the tumor cell. T cell activation and tumor cell killing only occurs in the presence of both antigens and spares healthy tissues that only express the primary antigen. **(C)**, Targeting of intracellular antigens by using genetically engineered TCR-T cells. Peptide-major histocompatibility complex (MHC) tumor-associated or tumor-specific antigens of mutated intracellular proteins are promising targets for the treatment of solid tumors. Tumor surface antigens are often expressed to some extent on healthy tissues and can cause severe off-tumor toxicity.

CAR-T cell therapies showed remarkable efficacy in certain B cell malignancies but struggle when it comes to myeloid malignancies and solid tumors. Challenges for CAR-T and TCR-T cell therapies that limit their clinical efficacy are severe adverse events, limited tumor infiltration, and persistence of genetically engineered T cells, as well as tumor immune evasion by loss of antigen. In particular, the complex nature of the immunosuppressive TME in solid tumors (112) is limiting T-cell infiltration and is promoting their exhaustion through presentation of inhibitory ligands on the tumor cells. There are a number of approaches with the aim to improve ACT by overcoming tumor antigen escape and increasing tumor specificity, and a selection of strategies will be highlighted here (**Figure 4**).

### 7.1 Adverse events associated with genetically engineered T cells

Immunotherapies with genetically engineered T cells are often accompanied by moderate to severe adverse events, limiting their clinical application in certain cases. The most common and severe adverse event of therapies with engineered T cells is cytokine release syndrome (CRS), which was first observed in clinical studies and did not occur in preclinical models at the time (113). Moreover, immune effector cell-associated neurotoxicity syndrome (ICANS), often referred as neurotoxicity, is very common in patients receiving CD19 CAR-T cells (114). Symptoms of CRS range from mild fever to life-threatening manifestations up to multi-organ system failure (115). Follow-up of patients receiving engineered T cells and monitoring for signs of CRS is vital to manage moderate to severe cases of CRS with anti-IL6 receptor agonist tocilizumab alone or in combination with corticosteroids, together with extensive supportive care (116). Symptoms of ICANS include headache, encephalopathy, tremor, and seizures, that are usually self-limiting, but rare lethal cases have been reported as well (117). Severe cases are often managed with corticosteroids, while tocilizumab is mostly ineffective in the treatment of ICANS, contrary to its effectiveness in CRS (116). Cross-talk between the engineered T cells and other immune cells, especially macrophages, can lead to the induction of systemic inflammation in the form of CRS which might cause leakiness of the blood brain barrier and symptoms of ICANS (116). Therefore, ICANS is often associated and correlates with the severity of CRS in patients, but it has also been reported in some cases in the absence of CRS (117).

On-target off-tumor toxicity is another challenge for adoptive T-cell therapies. In contrast to CRS and ICANS, this adverse event is not caused indirectly by associated endogenous immune cells but directly by the engineered T cells recognizing their cognate antigen or a cross-reactive antigen on healthy tissues. In particular, TCR-T cells warrant careful screening to avoid severe toxicity caused by autoreactivity due to lethal cases of tissue damage in the heart and brain in two early clinical trials targeting MAGE-A3 positive cancers (118, 119). As these TCRs were affinity enhanced, on-target off-tumor toxicity was likely increased. However, recent studies with affinity enhanced TCRs targeting MAGE-A4 showed clinical efficacy in the absence of severe TCR-T cell-mediated toxicity (120–122), indicating the necessity to investigate each single modified TCR for such risks although also non-modified TCRs have the potential for cross-reactivity. These cases highlight the difficulties of screening methods for TCR candidates to exclude potential common and individualized severe autoreactivity, which is highly difficult to be tested sufficiently at the preclinical level, but also indicate that TCRs have the potential to represent safe therapies with promising efficacy.

For the use of allogeneic CAR-T and TCR-T cells, a potential additional adverse event risk is allo-reactivity of allogeneic engineered T cells against foreign MHC molecules or minor histocompatibility antigens on host cells that can lead to GvHD. As described above, early clinical trials suggest that gene editing for the removal of the endogenous TCR is a viable strategy to avoid GvHD. Moreover, the use of allogeneic engineered T cells post-HCT, HLA-matching of the donor cells, and the use of non-αβ T cells are potential strategies (123). However, it remains to be seen how durable the persistence of allogeneic engineered T cells with these strategies is compared to their autologous counterparts, since elimination of the allogeneic T cells by the host immune system is a serious concern here.

Moreover, accompanying therapies, such as lymphodepletion prior to T-cell therapy, can add to hematological toxicities like cytopenia, which is a common adverse event with often unknown origin (26). Overall, this highlights the complex clinical landscape in regard to adverse events of CAR-T and TCR-T cell therapies and the need for better preclinical models to predict them early on (113).

### 7.2 Persistence of genetically engineered T cells

Persistence of CD19 CAR-T cells for up to 10 years has been reported in two patients with chronic lymphocytic leukemia after remission (124). While CD8^+^ CAR-T cells were abundantly found in the initial response, it was almost exclusively CD4^+^ CAR-T cells that were present during long-term remission. However, poor T cell persistence has been reported in many CAR-T (125) and TCR-T cell (97) clinical trials against a variety of tumor entities and is often likely the reason for limited clinical efficacy or relapses. Therefore, administration of lymphodepleting regimens is commonly performed prior to the engineered T-cell therapy to increase T cell persistence (26).

Preclinical studies observed less exhaustion and improved antitumor activity of CAR-T cells with PD-1 knock-out (126, 127). Therefore, PD-1 knock-out is currently explored for the treatment of tumors with CAR-T (108, 125) and TCR-T cells (70) as a strategy to protect the engineered T cells from exhaustion and to enhance their persistence. Stadtmauer et al. used CRISPR-Cas9 genome editing to remove the endogenous TCR and PD-1 and introduced an engineered TCR specific for the cancer-testis antigens (CTAs) NY-ESO-1 and LAGE-1 to treat two patients with refractory melanoma and one with sarcoma. Engineered T cells trafficked to the sites of the tumor and reduction of the target antigens, likely as a response to the immune pressure of the TCR-T cells, was observed for both melanoma patients. Interestingly, no toxicity was observed and persistence of the TCR-T cells was increased in all three patients with at least 9 months compared to previous trials with T cells that retained their endogenous TCR and PD-1 expression. However, the number of patients in this first-in-human phase I clinical trial is low (NCT03399448) and expansion of the study is necessary. It also remains to be elucidated if the prolonged persistence is based on the ablation of PD-1 or in part on the removal of the endogenous TCR. Moreover, PD-1 ablation was also reported to cause increased functional exhaustion and cell death along greater activation in a CD19 CAR-T cell therapy (128). Results from knock-out experiments of PD-1 in mice with a chronic lymphocytic choriomeningitis virus (LCMV) infection showed that CD8^+^ T cell exhaustion can not only occur in the absence of PD-1 but PD-1 even protected the cells from overstimulation and terminal differentiation to an exhausted effector phenotype at the site of infection (129). Suggesting that PD-1 could be relevant to fine-tune T cell responses in certain environments, such as high antigen load as is the case in viral infections. In that context, transient blockade of PD-1 with ICI could be superior over PD-1 ablation but our understanding how PD-1 signaling modulates gene expression during T cell responses remains enigmatic and needs to be further elucidated. A recent study showed that genes associated with survival and proliferation are resistant to PD-1-mediated inhibition while effector functions are regulated by it based on the TCR signal strength (130). Due to the context-dependent functions of PD-1 signaling, it remains to be seen if PD-1 ablation of genetically engineered T cells is an effective way of improving T cell persistence depending on the tumor entity and antigen load.

Moreover, a number of costimulatory switch receptors have been reported to prevent exhaustion of genetically engineered T cells and might increase their persistence (131–133). Switch receptors consist of the extracellular portion of an inhibitory receptor (e.g. PD-1, TIGIT, TIM-3) and the intracellular signaling domain of a costimulatory receptor (e.g. CD28, 4-1BB). Reports from the preclinical studies are encouraging with improved antitumor activity and persistence of genetically engineered T cells armored with these switch receptors, however, these signaling axes are complicated in nature. Tipping the scale from exhaustion to activation and not just balancing it as is the case with anti-PD-1/PD-L1 ICI might lead to overstimulation and a dysfunctional T cell phenotype.

Tonic endogenous TCR signaling was also associated with improved persistence of CAR-T cells in recent studies (125, 134). Removal of the endogenous TCR and PD-1 in mesothelin (MPTK)-specific CAR-T cells for the treatment of solid tumors resulted in poor persistence of the TCR-deficient CAR-T cells beyond 6 weeks in a phase I clinical trial with 15 patients (NCT03545815 (125)). Surprisingly, it was the TCR-positive CAR-T cells that became the main fraction after infusion in three patients despite their rare presence in the infused cell product. The authors replicated these findings in mice and hypothesized that tonic TCR signaling plays a beneficial role in CAR-T cell persistence. Off note, this was in the scenario of low-level engraftment and might be different when using lymphodepletion to increase engraftment. In line with these observations, another study also observed reduced persistence for TCR-deficient CD19 CAR-T cells in animal models (134). The role of tonic TCR signaling for the longevity of CAR-T cells (19) needs to be further addressed especially in the context of allogenic CAR-T cell therapies, where the removal of the endogenous TCR is already common practice to prevent GvHD (108, 109). TCR-T cells with endogenous TCR replacement are likely not affected due to tonic signaling of the introduced TCR as indicated by the results from Statdmauer et al.

### 7.3 Potential strategies to overcome tumor antigen escape

A common form of tumor resistance to ACT is tumor antigen escape by loss or downregulation of surface antigens or peptide-HLA complexes (135). Targeting multiple antigens by engineering T cells with a dual CAR or a tandem CAR could reduce the risk of tumor antigen escape (**Figure 4A**). Preliminary results from a phase I study (NCT03233854) with a CD19/CD22 bispecific CAR showed clinical efficacy but antigen escape in relapses was mostly observed for CD19 and not CD22 antigen, suggesting that there was less immune pressure of the CAR-T cells on the CD22 target (136). This is supported by the observation that CD22 scFV ligation in the bispecific CAR showed less cytokine secretion than for CD19 scFV. Another bispecific CD19/CD22 CAR that uses a tandem approach showed good efficacy in 6 patients with r/r B-ALL and observed one relapse of blast cells with loss of CD19 antigen and diminished CD22 expression 5 months after treatment (NCT03185494 (137)). The single construct approach for multi-specific CAR-T cells might affect the antigen binding capabilities based on the design of the linkers and it could be favorable to use a bi- or tricistronic design that expresses individual CAR molecules on the same cell to avoid these problems. This has been done for tricistronic CD19/CD20/CD22 tri-specific CAR-T cells that were able to target B-lineage ALL independent of CD19 expression *in vitro* and in animal models (138). Results from a phase I study (NCT03289455) with bicistronic CD19/CD22 bispecific CAR-T cells for the treatment of 15 patients with r/r B-ALL showed 86% CR and one-year overall survival and event-free survival of 60% and 32%, respectively (139). Dual targeting of B7-H3 and CD70, which are overexpressed in a variety of solid tumors, with a tandem CAR elicited superior tumor control and overall survival in a lung cancer and melanoma xenograft model (140). Moreover, infusing patients with a mixture of mono-specific CAR-T cells or TCR-T cells with different specificity could also be a viable option and has recently been done for patients with solid tumors that received up to three neoTCR-T cells with different specificity (97).

Multi-targeting of different antigens seems to be viable strategy to overcome tumor antigen escape and preliminary results suggest that it might increase antitumor immunity against tumor cells that co-express multiple antigens. However, this approach is limited so far by the number of known promising tumor antigens and will benefit from the discovery of additional tumor-associated and tumor specific antigens in the future.

### 7.4 Potential strategies for the treatment of solid tumors

The treatment of solid tumors is one of the most difficult areas in the field. Limited T-cell infiltration into the tumor as well as T cell exhaustion due to the immunosuppressive TME poses a high risk for an insufficient response to the treatment. In addition, the heterogeneity of antigen expression and the lack of truly tumor-specific surface antigens in solid tumors (112) can cause severe off-tumor toxicity in healthy tissues. Due to the difficulty of identifying tumor-specific surface antigens on solid tumors as targets for CAR-T cell therapies, strategies with higher specificity and less off-tumor toxicity have been developed, such as the synthetic Notch (synNotch) receptor designs (**Figure 4B**). Recognition of a primary antigen by the synNotch receptor cleaves an orthogonal transcription factor from the cytoplasmatic tail and induces the expression of a CAR that can recognize a secondary antigen on the tumor cell (141). T cell activation and tumor cell killing only occurs if both antigens are expressed on the tumor cell. This concept has been applied in a number of preclinical studies for solid tumor models and demonstrated improved specificity for the treatment of solid tumors (142, 143). SynNotch circuits can also be used to improve the specificity of engineered TCRs for selective killing of tumor cells, which has been demonstrated for a SynNotch-TCR against melanoma cells *in vitro* (144).

TCR-T cells are inherently equipped for the recognition of intracellular antigens through peptide-HLA presentation which opens up a treasure trove of tumor-associated or tumor-specific antigens that could be exploited for TCR-T cell therapy of solid tumors (**Figure 4C**). As mentioned above, a preliminary study showed the feasibility and efficacy of TCR-T cells in two melanoma and one sarcoma patient (70) and clinical trials for the treatment of MAGE-A4 positive solid tumors showed clinical efficacy in subset of patients with an ORR of 24% (9/38) (121). Foy et al. demonstrated recently the feasibility of TCR-T cell therapy targeting personalized neoantigens (97). A combinatorial screening approach with whole exome sequencing (WES) and RNA sequencing of the patients’ tumors was used to predict potential neoantigens. In a next step, multimeric labeled peptide-HLA complexes were generated for reactivity assessment of the predicted neoantigens in peripheral blood of patients and neoTCRs were identified and manufactured for 16 patients. Despite this impressive demonstration of feasibility of such a complex workflow in a clinical setting, efficacy and T cell persistence was limited. However, there are over a hundred clinical trials with adoptive TCR-T cell transfer registered in clinicaltrials.gov with the majority of them for the treatment of solid tumors (145). Hurdles for TCR-T cell therapy include its dependency on a specific HLA genotype, restricting it to a specific patient population in most cases, and its susceptibility to HLA-downregulation. Moreover, intracellular antigens can also be targeted with TCR-like CARs that use scFV molecules that recognize specific peptide-MHC complexes. Recently, a phase I clinical trial with a TCR-like CAR T-cell therapy targeting MAGE-A4 peptide-HLA-A*02:01 complexes for the treatment of solid tumors has been initiated (146) and evaluation of the clinical efficacy of more TCR-like CAR formats in clinical trials will be of great interest for the use of this concept. Overall, combinatorial approaches with a variety of different interventions will likely be necessary for the treatment of solid tumors in the future in order to overcome their complex mechanisms of immune evasion.

## 8 Future perspective

Adoptive immunotherapies with genetically engineered T cells for the treatment of refractory tumors is a new breakthrough therapy with promising efficacy in certain cancers. However, its application is held back by manufacturing difficulties, severe adverse events, regulatory challenges, and extremely high costs. There are a number of strategies how limitations are currently addressed. Automated and expedited manufacturing processes might have an impact on both product quality as well as costs. Approaches with allogeneic donor T cells may improve availability but also provide “off-the shelf” therapies and therefore substantially result in cost reduction. Due to the HLA-restriction of TCR-T cell therapies, it seems unlikely that companies will go through the expensive process of testing this type of therapy in tumor types where CAR-T cell therapies are already showing good efficacy at this point. Therefore, TCR-T cells are mostly tested in solid tumors where the lack of good surface targets limit CAR-T cell therapy. At the moment, TCR-T cells are usually targeting tumor-associated antigens but first tumor-specific studies have demonstrated their feasibility and personalized approaches might be more prevalent in the future. Improving tools to predict potential on-target off-tumor toxicity is especially crucial for TCR-T cells due to their higher antigen sensitivity compared to CAR-T cells, especially for affinity enhanced TCRs. This is in particular important for time-saving clinical translation of newly identified, optimized or personalized TCR constructs. Additionally, the development of novel preclinical models for the prediction of associated adverse events like CRS and ICANS is necessary to develop novel strategies to exclude most severe adverse events before clinical testing in the future (113). The number of clinical studies exploring new strategies and the speed the field is innovated upon is impressive. However, it is difficult for the regulation of these novel and complex therapies to keep up with this speed, and standardization of certain manufacturing steps will likely be necessary to ensure safety and comparability of T cell products for patients in the future. To make these therapies more commonly available and explore their benefit not only for refractory tumors but also at earlier stages of disease, their costs and resources for manufacturing must become more sustainable for the health care system (60, 147).

One of the major challenges is the development of novel strategies in case of resistance. Personalized combinatorial approaches to target multiple antigens and to neutralize the immunosuppressive TME probably will be necessary for the treatment of solid tumors but also resistant hematological malignancies in the future.

While ACT with genetically engineered T cells is mainly used as a therapy for cancer, its potential for the treatment of other diseases is more and more realized. Preliminary results of CD19 CAR-T cell therapy in patients with refractory systemic lupus erythematosus (SLE) showed that it was well tolerated and highly effective (148). Exploring its use for other autoimmune but also genetic diseases might open up effective novel options for treatment failures in the future.

## Author contributions

MH conceptualization, writing, drawing of figures, review and editing. AK conceptualization, writing, review and editing. All authors contributed to the article and approved the submitted version.
